# Assessment of a probabilistic framework for combining structure- and ligand-based virtual screening

**DOI:** 10.1186/1758-2946-4-S1-P7

**Published:** 2012-05-01

**Authors:** Simone Fulle, Stuart M Armstrong, Paul W Finn, Garrett M Morris

**Affiliations:** 1 InhibOx, Limited, Oxford, UK

## 

A wide variety of structure- and ligand-based virtual screening approaches have been developed that aim at finding potential leads to initiate drug discovery efforts. Since each method has its strengths and weakness, combining the outcome of different structure- and ligand-based approaches can be expected to decrease the number of false positive predictions. However, a reliable fusion of information from different methods is challenging. This holds true in particular for new target structures, where target specific performance experiences are missing.

Here, we assess the performance of a probilistic framework approach [1] that combines structure- and ligand-based information in a meaningful way by assigning probabilities that any two molecules are active. The approach is validated using two popular docking methods (GOLD and AutoDock) and an in-house ligand-based screening approach (ElectroShape [2]). Results of similarity search and docking calculations for the Directory of Useful Decoys (DUD) [3] are combined through rank fusion as well as a probabilistic framework approach.

The study will be used to answer questions such as: How far do the virtual screening-approaches used provide complementary or redundant hit lists? Does the fusion of structure- and ligand-based approaches consistently outperform any single screening metric? Using a probabilistic framework approach, is it possible to obtain a quantification of the confidence that any molecule will be active?
